# GPR91: expanding the frontiers of Krebs cycle intermediates

**DOI:** 10.1186/s12964-016-0126-1

**Published:** 2016-01-12

**Authors:** Matheus de Castro Fonseca, Carla J. Aguiar, Joao Antônio da Rocha Franco, Rafael N. Gingold, M. Fatima Leite

**Affiliations:** Department of Physiology and Biophysics, Federal University of Minas Gerais, Av. Antonio Carlos 6627, Belo Horizonte, MG CEP: 31270-901 Brazil; Centro Universitário Estácio de Sá, Belo Horizonte, MG Brazil

**Keywords:** Succinate, GPR91, Cell functions, Cell signaling

## Abstract

Since it was discovered, the citric acid cycle has been known to be central to cell metabolism and energy homeostasis. Mainly found in the mitochondrial matrix, some of the intermediates of the Krebs cycle are also present in the blood stream. Currently, there are several reports that indicate functional roles for Krebs intermediates out of its cycle. Succinate, for instance, acts as an extracellular ligand by binding to a G-protein coupled receptor, known as GPR91, expressed in kidney, liver, heart, retinal cells and possibly many other tissues, leading to a wide array of physiological and pathological effects. Through GPR91, succinate is involved in functions such as regulation of blood pressure, inhibition of lipolysis in white adipose tissue, development of retinal vascularization, cardiac hypertrophy and activation of stellate hepatic cells by ischemic hepatocytes. Along the current review, these new effects of succinate through GPR91 will be explored and discussed.

## Background

Back in the 1920’s, succinate (succinic acid in blood pH), a dicarboxylic acid, was firstly correlated to a carbohydrate oxidation sequence proposed by Thorsten Thunberg, 1920 [1]. In the following decade, this sequence of oxidation was better described thanks to Albert von Szent-Györgyi’s studies on pigeon breast muscle [2]. Consequently, succinate’s catalytic role as a hydrogen carrier in aerobic respiration was discovered [2]. Then, in the late 1930’s, Krebs described the core of aerobic respiration, the Krebs cycle – also referred to as tricarboxylic acid cycle or citric acid cycle [3]. With a few posterior details added, this cycle remains the best description of aerobic respiration to date [4]. Thereafter, for many decades succinate had been considered only as an intermediate of Krebs cycle, which was thought to be its single synthesis pathway.

**Fig. 1:**
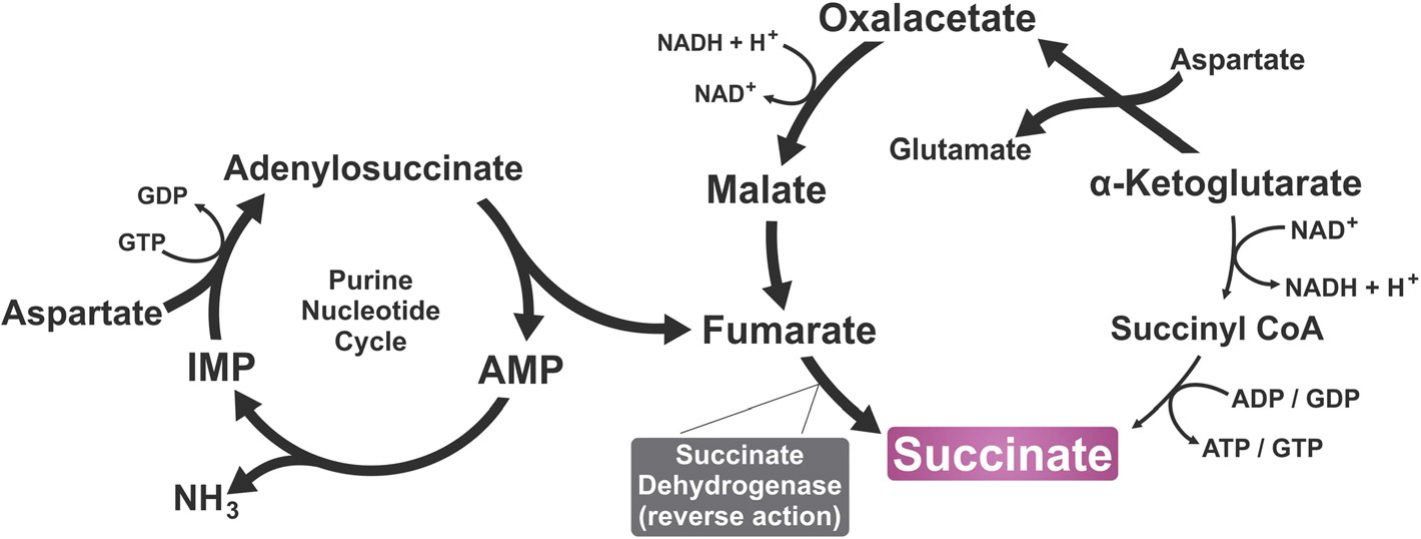
Pathway of succinate accumulation during ischemia/reperfusion. Despite minor production via regular Krebs cycle - which is diminished due to excessive synthesis of NADH -, the reverse activity of succinate dehydrogenase has been shown to be the leading cause of succinate increase during ischemia. The sources of fumarate, which is then reduced into succinate, are mainly the Purine Nucleotide cycle – shown on the left - and the Malate/Aspartate Shuttle – similar to the Krebs cycle to run in reverse, which is favored by high levels of NADH

Recent studies, however, demonstrated that succinate could be produced in nonenzymatic manners as well. This is possible because, under oxidative stress conditions, in which some enzymes of the Krebs cycle are inhibited, α-ketoglutarate levels are alternatively generated via transamination. Its accumulation combined with inactivation of α-ketoglutarate dehydrogenase – from the Krebs cycle – leads to nonezymatic decarboxylation of α-ketoglutarate into succinate [5]. It is noteworthy that in 1970, Krebs noticed that some of the Krebs’ metabolites, including succinate, could accumulate in the interstitial space in case of ischemia, although the mechanisms and metabolic implications were not completely explained at the time [6]. Recently, Chouchani and colleagues (2014) described a mechanism by which extracellular concentration of succinate augments in case of ischemia (Fig. 1). ^13^C-isotopologue labelling assessments indicated that succinate was not generated by the common sources used in normoxia, which include glucose, glutamate, fatty acids and GABA (γ-aminobutyric acid) shunt. Moreover, these authors found that infusions of dimethyl malonate, the precursor of malonate – a competitive inhibitor of succinate dehydrogenase – caused decrease in succinate accumulation, altogether demonstrating that succinate increases during ischemia is due to succinate dehydrogenase reverse action, reducing fumarate – generated from malate/aspartate shuttle and the purine nucleotide cycle, since both this pathways are favored by ischemic conditions - into succinate [7, 8], (Fig. 1). Thus, succinate is an important intermediate metabolite of the citric acid cycle that can be generated by different pathways within the mitochondria. In conditions linked to insufficient blood supply, such as ischemia, succinate is generated through a pathway distinct from Kreb’s cycle, and its concentration at blood vessels might rise.

Hence, once accumulated within the mitochondrial matrix, succinate can migrate to the cytosol through dicarboxylate transporters located in the inner mitochondrial membrane. SLC25A10 (solute carrier family 25 member 10), a succinate-fumarate/malate transporter, is considered hitherto the responsible for this translocation. The second phase of transport, in which succinate crosses the outer mitochondrial membrane, is thought to happen by means of porins, namely, proteic channels through which various nonspecific molecules of less than 1.5 kDa may pass. Lastly, succinate has a fast efflux system into the bloodstream. The transporter, a protein named INDY (for I’m not dead yet, on account of its apparent relation to longevity), is a sodium-independent anion exchanger [9] (though previous studies considered it a sodium-coupled transporter) [10], which is able to switch dicarboxylates and citrate across the cell membrane.

Why are these alternative ways of succinate synthesis and its transport mechanism relevant? Mainly, because of the several functions attributed to succinate other than participating in the Krebs cycle, some of them – the ones on which this paper will focus – are associated with a G protein-coupled receptor, known as GPR91or SUCNR1, which has succinate as its specific ligand [11]. Upon binding to such receptor, succinate has a hormone-like function acting in various organs and tissues such as blood cells, adipose tissue, liver, heart, retina, and kidneys – being expressed the most in these latter [11].

## An overview on GPR91 and its expression pattern

**Fig. 2:**
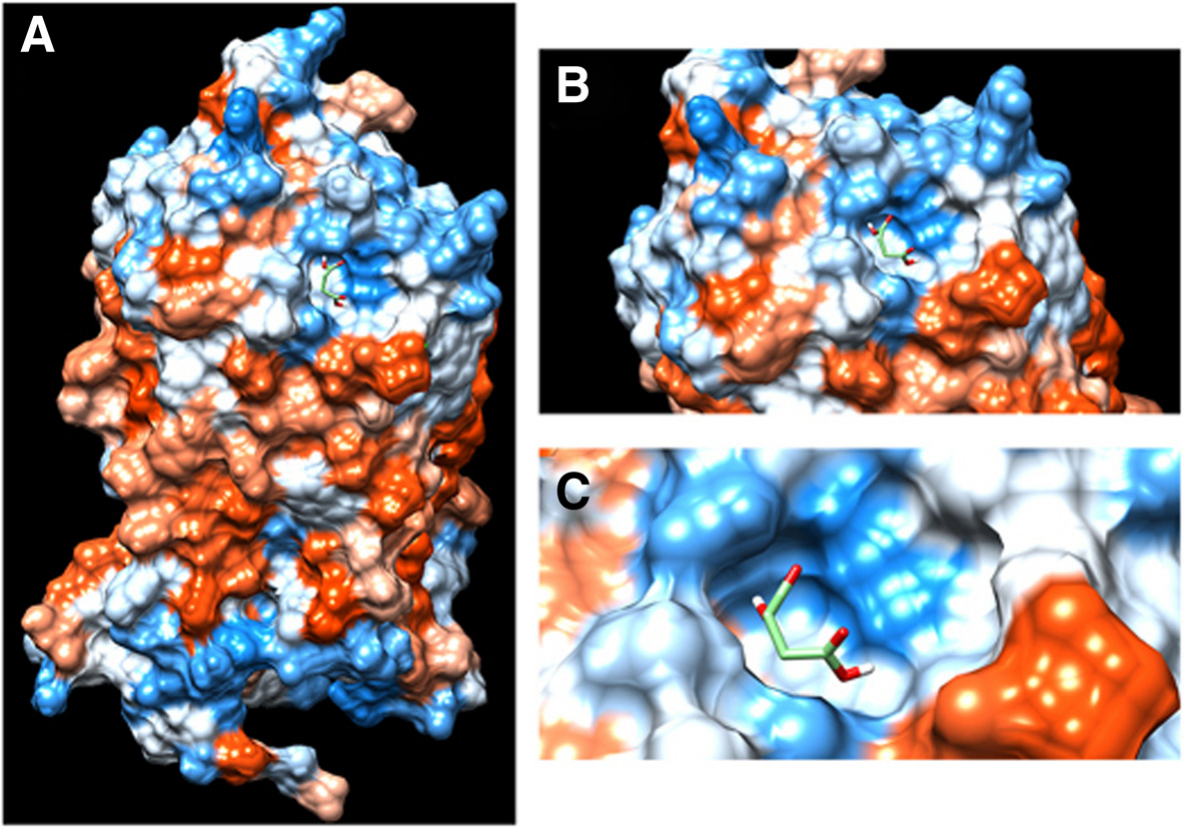
Schematic model of the GPR91 active site. Surface representation of succinate binding at the active site with electrostatic potential (red, blue for negative and positive potential, respectively) computed with the GPR91 tool in Website Protein Data Bank (PDB). **a** through **c** represent consecutive higher magnifications of the succinate binding site on GPR91

GPR91 is a G-protein coupled receptor that acts as a sensor of extracellular succinate [11; reviewed in 12]. When it comes to the receptor structure, mutation experiments demonstrated that Arg^99^, His^103^, Arg^252^ and Arg^281^ play an important role in receptor function. These amino acids are all located in helices and agglomerate in the central area of the receptor - a rhodopsin-like structure **-** in a way that the positive charge attracts succinate [11]. We now show the result of an *in-silico* study of GRP91 and its possible succinate-binding site (Fig. 2). Although GPR91 is 33 % homologous to GPR99, a receptor linked to α-ketoglutarate, affinity assays have shown that succinate binds exclusively to GPR91, while α-ketoglutarate is a ligand for GPR99 [11]. In fact, the EC_50_ values regarding succinate-GPR91 stimulation range from 20 to 50 μm [11]. To test GPR91 ligand binding affinity, several substances, including pharmacological compounds for different GPCRs, and carboxylic acids close to succinate were tested. Some of them could also bind to GPR91, but with a much lower affinity compared to succinate [11–13]. Thus, it is now well accepted that succinate is the endogenous ligand for GPR91.

GPR91 interacts with multiple G-proteins. According to some studies using pertussis toxin, GPR91 can couple either with G_i_ or G_q_, triggering different pathways and initiating distinct cellular effects. In HEK293 and MDCK (kidney derived cells), for example, succinate induces intracellular calcium release, inositol triphosphate formation, extracellular-signal-regulated kinases 1/2 (ERK1/2) activation and decrease of cyclic adenosine monophosphate (cAMP) concentration, which are signaling pathways induced by G_q_ or G_i_ coupling, depending only on succinate concentration [11]. In hematopoietic progenitor cells, however, signaling mediated exclusively by G_i/o_ leads to proliferation due to ERK1/2 activation [14]. In cardiomyocytes, succinate increases rather than decreases cAMP, leading to protein kinase A (PKA) activation, and suggesting that GPR91 coupling to Gs is also possible [15]. These distinct intracellular signaling pathways initiated by GPR91 activation indicate that succinate actions as a hormone can indeed be very diverse. Moreover, after triggering the signal transduction cascade, GPR91 is known to undergo internalization. Imaging studies indicated that GPR91 is located specifically on the plasma membrane, and is internalized and then desensitized as a result of ligand stimulation [11; reviewed in 12].

Although, GPR91 was initially characterized in the kidney, and shown to be highly expressed in liver, spleen and intestine [11], GPR91 is now known to be present throughout the body, including a variety of excitable as well as non-excitable cells. In the kidney, GPR91 localizes to the renal vascular lumen, in particular the afferent arteriole and the glomerular vasculature. Moreover, GPR91 is expressed in the luminal membrane of multiple segments of the renal tubules: the cortical thick ascending limb (CTAL) of Henle’s loop, including the apical membrane of macula densa (MD), and the cortical and medullary collecting duct (CD) [16–18], but renin-producing juxtaglomerular cells (JGA), mesangial cells, and vascular smooth muscle cells that are key components of the JGA were found to be GPR91 negative [17]. In the liver, GPR91 is exclusively expressed in quiescent hepatic stellate cells (HSCs) [19], while in the heart ventricular cardiomyocytes express GPR91 in the sarcolemma membrane and T-tubules [15]. In the retina, GPR91 is predominantly expressed in the cell bodies of the retinal ganglion cell (RGC) layer [20]. White adipocytes, hematopoietic progenitor cells [21] and multiple types of blood and immune cells were reported to express GPR91 [14, 22]. GPR91 was also detected in immature dendritic cells (DCs). Thus, since its characterization as the receptor for succinate in 2004 [11], GPR91 has been described in many cell types, and demonstrated to have a vast array of functions in the human body. Further details regarding the role of succinate through GPR91 in some of the aforementioned systems will be discussed below.

## GPR91 signaling in the liver

**Fig. 3:**
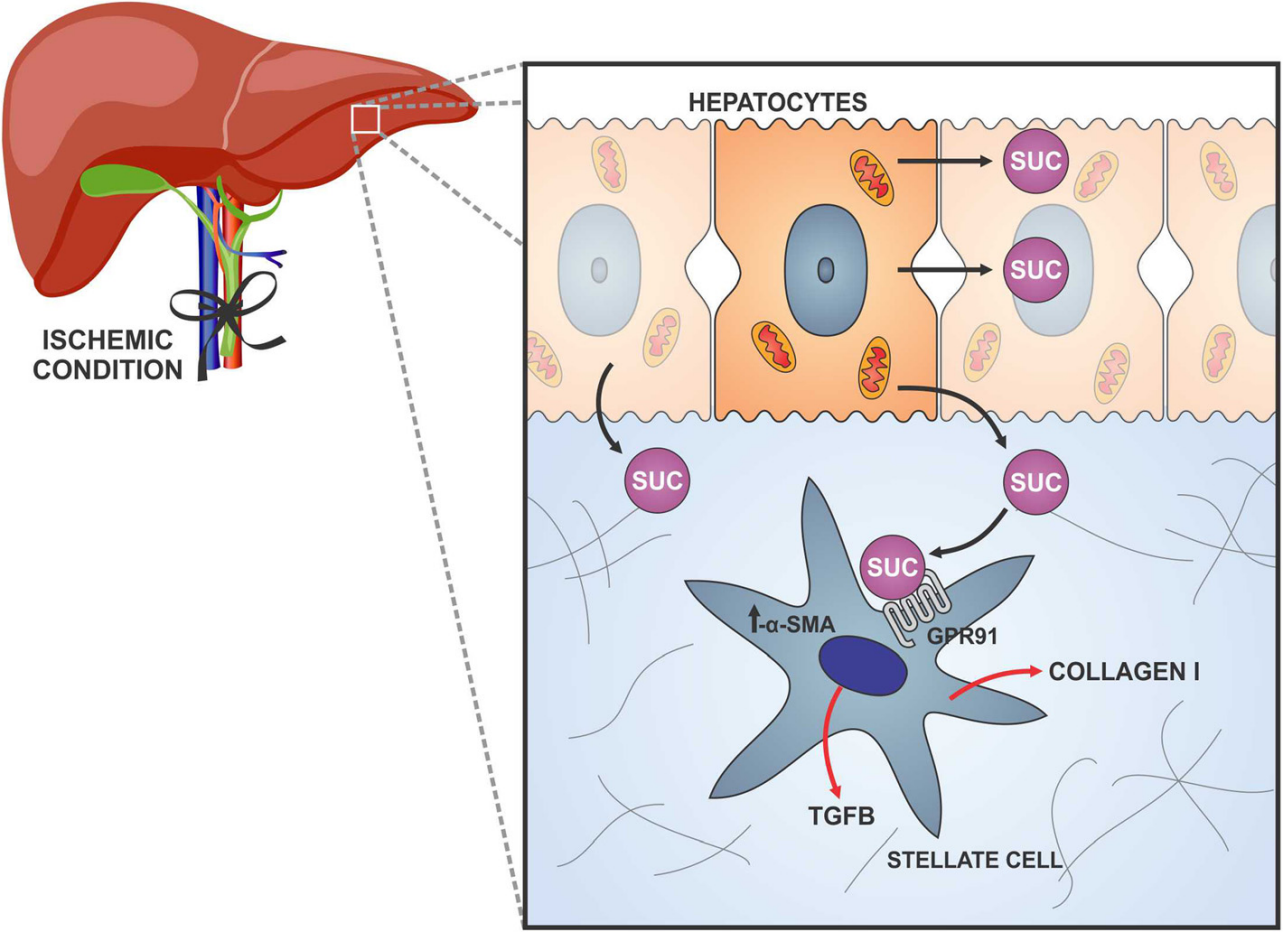
Succinate role in the liver through GPR91. During an ischemic condition, succinate is released by anoxic hepatocytes and binds to stellate cells leading to their activation. Once activated, stellate cells increase expression of several fibrogenic markers, such as alpha-smooth muscle actin (α-sma), TGF-B and collagen type I

The liver is targeted by a high number of growth factors and hormones that bind directly to hepatocytes or to other cell types such as HSCs. Many of these molecules (PDGF and TGF-β, for example) activate stellate cells during liver damage. Also, non-traditional signals such as matrix stiffness, metabolites and oxidative stress [23] are able to activate HSCs. A study published in 2007 by Correa and colleagues suggesting that succinate may behave as a metabolic sensor in the liver, has extended our comprehension of how liver injury can stimulate succinate production and consequent HSC activation [19]. According to Correa et al., 2007, *in vitro* ischemia-reperfusion in rodent livers, allowed detection of succinate in extracellular fluids, and this phenomenon played an important role in the activation of HSCs (Fig. 3). The same group showed that, in the liver, GPR91 is expressed primarily in the quiescent stellate cell and this expression is lost when stellate cells are activated. On the other hand, Li et al., 2015 showed that in the presence of succinate, HSCs present an increased GPR91 expression followed by a 2-fold increase in the differentiation rate, thus leading o their activation [24].

In addition, Li and colleagues 2015, expanded the previous finding, by demonstrating the specific molecular effects of succinate in the activation of HSCs. On a study using *in vitro* and *in vivo* models, it was shown that HSCs cultured and treated directly with succinate or with inhibitors of succinate dehydrogenase (malonate, palmitate/choline and methionine-choline deficient media), caused not only an increase in expression of GPR91, but also of alpha-smooth muscle actin (α-sma), TGF-B and collagen type I, markers of a fibrogenic response [24] (Fig. 3). On the other hand, transfection of these cells with siRNA for GPR91 abrogated α-sma production induced by succinate, indicating the specificity of the pro-fibrogenic responses to GPR91 signaling. In the *in vivo* studies, HSCs isolated from hepatosteatotic mice, fed with a methionine and choline deficient diet, released a higher level of succinate and expressed more GPR91 and α-sma as well. Taking together, these findings indicate a succinate-GPR91 coupling dependence in HSCs activation and fibrogenesis. Therefore, GPR91 might play a relevant role in liver homeostasis, being a possible therapeutic target for modulation of hepatic functions. Blocking GPR91 during liver transplantation could, for instance, avoid undesirable fibrogenic reactions.

## GPR91 signaling in retina

**Fig. 4:**
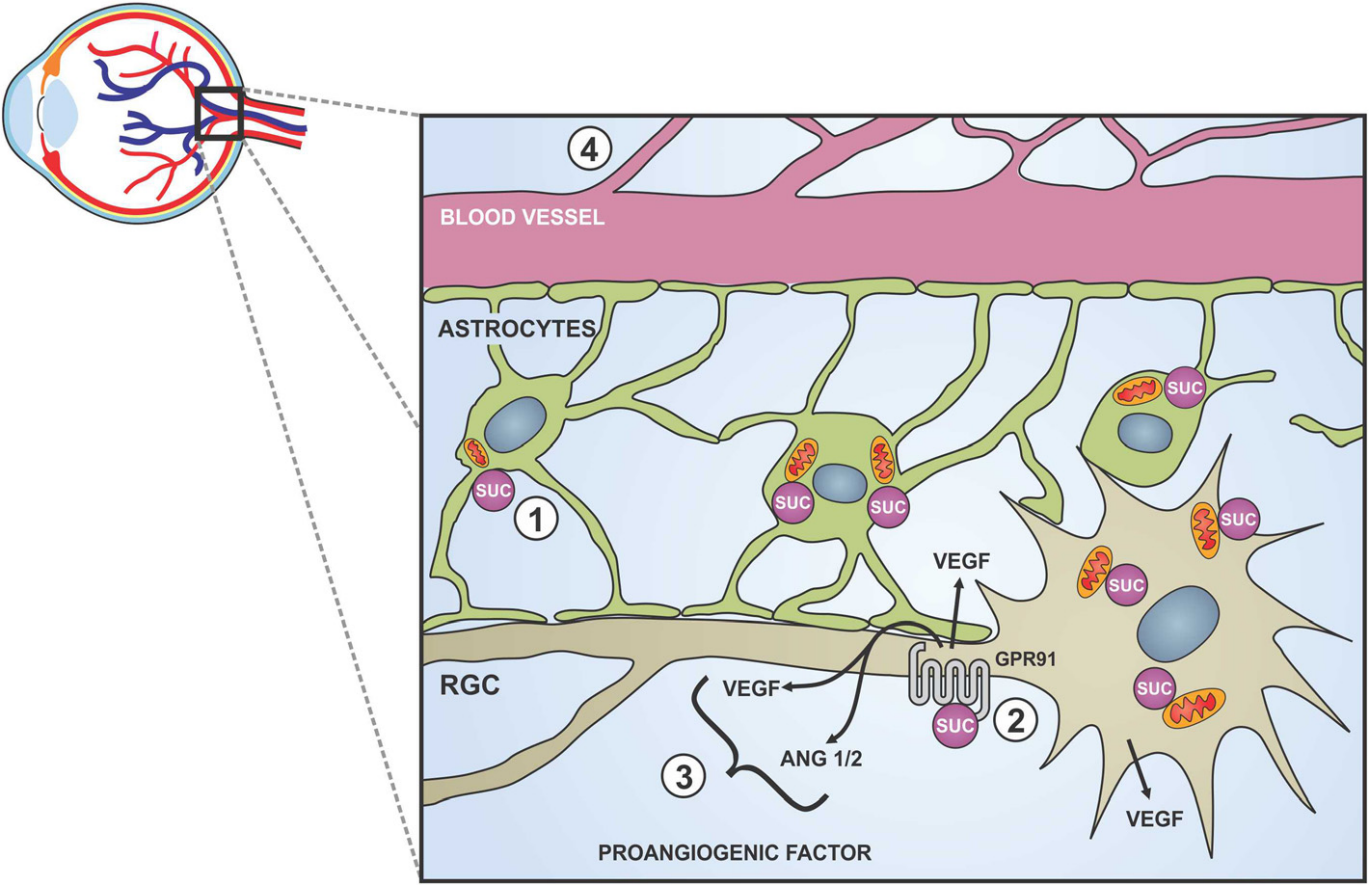
GPR91 activation induces retinal neovascularization in ischemic proliferative retinopathy. Under normal conditions, retina presents a basal vascularization. (1) During hypoxia, succinate accumulates and (2) binds to GPR91 on RGCs, leading to (3) proangiogenic factor production that stimulate the development of new vessels (4) in order to restore the vascular supply to the hypoxic retina

Besides liver injury, succinate effects through GPR91 activation have been demonstrated in a very delicate and well-specialized structure such as retina, which owns a vast vascular plexus responsible for its metabolic requirements [25]. In certain conditions, a misbalance between tissue demand for oxygen and nutrients and vascular supply results in hypoxic retina, leading to a detrimental preretinal and intravitreal neovascularization. Since vascular supply is coupled to tissue metabolic rate, and succinate accumulates under conditions of insufficient oxygen supply [26, 27], the role of succinate in retinal neovascularization induced by hypoxia was investigated. It was found that in ischemic retinas of rats subjected to oxygen-induced retinopathy, succinate levels rise without a joint increased expression of GPR91, which maintained its levels in retinal ganglion neurons (the main retinal structure where GPR91 is localized) [20]. In addition, succinate induced a prominent development in retinal vascularization and vascular density, an effect significantly suppressed by siRNA to GPR91. In this report, Sapieha and colleagues, 2008, showed that the role of succinate in this system is to induce an autocrine activation of retinal ganglion neurons by its binding to GPR91. Therefore, in response to augmented succinate levels, these cells regulate the production of an array of angiogenic factors including the vascular endothelial growth factor (VEGF) through a specific activation of GPR91. Of note, there is no participation of a hypoxia-inducible factor-1a (HIF-1a)-dependent pathway (Fig. 4). Accordingly, succinate showed no effect on rats that are deficient in retinal ganglion neurons, confirming the importance of these cells in orchestrating GPR91-succinate-dependent neovascularization. Conversely to succinate effects, Semaphorin 3A (a class of secreted and membrane protein that acts as an axonal growth cone guidance molecule) has been suggested as an opposite force to neovascularization [28]. Joyal et al., 2011, suggested that this molecule promotes vascular decay and later forms a chemical barrier that repels neo-vessels toward the vitreous [28].

Adding on the succinate effects on retinal neovascularization described above, Hu et al., 2013 have shown that GPR91 also regulates VEGF production in the retinal cell line RGC-5 through its direct incubation with succinate or a high-glucose medium [29]. In addition, ERK1/2 and JNK signaling pathways may be involved in the effects of GPR91-mediated high glucose-induced VEGF release in RGC-5 cells. A very recent work from the same group reinforced these findings. Using RGC-5 cells, Hu et al., 2015, demonstrated that GPR91 mediates VEGF secretion and endothelial cell proliferation, possibly by activating the ERK1/2 and JNK signaling pathways and then upregulating COX-2 (Cyclooxygenase 2) and PGE2 (prostaglandin E2) expression [30]. Since COX-2 gene encodes a cytosolic protein that is upregulated throughout inflammation and may contribute to local ischemia and hypoxia, a higher COX-2 expression suggests that inflammation has an important part in the incidence and evolution of diabetic retinopathy [30].

In summary, strong evidence has put succinate/GPR91 as an important signaling pathway to activate the development of new blood vessels in retina during a hypoxic condition and modulation of VEGF release through this process might be a possible target for therapy.

## GPR91 signaling and metabolism

The effects of GPR91 signaling pathways on metabolism were first demonstrated by Sadagopan, 2007. By using rodent models of diabetes, obesity and hypertension, it was demonstrated that circulating succinate levels in these animals are elevated compared to non-diseased controls [31]. However, the mechanisms that lead to this increase in succinate concentration remain unclear. Contrary to what was observed in rodents, neither hypertension nor diabetes was associated with a rise in circulating succinate in human blood samples [31]. While these observed differences were not elucidated yet, another study using blood samples from patients that underwent liver transplantation showed increased levels of succinate as early as 2 h post-transplant, which was also observed 6 h after transplant [15].

More recently, McCreath and colleagues, 2015, showed that GPR91 is vastly expressed in the white adipose tissue of mice and regulates adipose mass and glucose homeostasis [32]. By generating a GPR91mutant mouse, it was shown that loss of succinate receptor leads to dichotomous effects on metabolism and total body weight, with no difference of weight among organs, but with a marked difference on the cumulative fat content [32]. On a regular diet, Sucnr1-/- mice present a smaller white adipose tissue compartment, smaller adipocytes, increased energy expenditure and an improved glucose buffering. Although it could be thought that decreased expression of GPR91 would lead to a reduced expression of genes related to adipocyte differentiation, deletion of GPR91 did not alter adipogenesis but rather resulted in diminished lipid accumulation and smaller adipocyte size. These results were further evaluated by the VO2 test, to investigate the metabolic changes caused by GPR91 deletion. As expected, the rate of VO2 consumption was reduced in Sucnr1-/- mice compared to WT counterparts. In contrast, feeding Sucnr1-/- mice with a high fat diet leads to increased fat deposition, hyperglycemia, reduced insulin secretion and an augmented hepatocyte damage compared to wild-type (WT) littermates. Thereby, these findings put GPR91 as a sensor for dietary energy, being a possible target for therapeutics on obesity, hypertension and diabetes.

## GPR91 signaling in the heart

Succinate is an important molecule for the maintenance or disturbance of cardiovascular status, specially blood pressure and cardiac muscle thickness. In 2010, Aguiar and collaborators showed that GPR91 is expressed in ventricular cardiomyocytes, in the sarcolemmal membrane, and in T-Tubules [33]. Because the GPR91 receptor is expressed in cardiomyocytes, and cardiac function is an important determinant of blood pressure and other aspects of cardiovascular homeostasis, there was an urge to investigate the role of succinate through cardiac GPR91. Aguiar et al. in 2010 showed that succinate could directly activate GPR91 in cardiomyocytes, affecting the pattern of intracellular Ca^2+^ release and Ca^2+^ re-uptake. It was demonstrated that proteins such as phospholamban and ryanodine receptor, both well known to be involved in the dynamic of cardiomyocyte Ca^2+^ release, are activated by succinate/GPR91 interaction. These effects were triggered by adenylyl cyclase and consequently PKA activation, resulting in cardiomyocyte apoptosis [33], an event that was prevented by PKA inhibitor. Together, these results confirmed that succinate could indeed, at high concentration, lead to cardiomyocyte cell death. Moreover, these data suggested that increased levels in serum succinate, which could be triggered for instance in ischemic conditions, might represent an important cause of cell death in the heart.

Furthermore, in 2014 it was demonstrated that long-term exposure to succinate could lead to cardiomyocyte hypertrophy [15]. Since activation of GPR91 in kidney can increase blood pressure through the renin-angiotensin system (RAS) [11], the question remained whether cardiac hypertrophy, induced by high levels of succinate in the blood stream, was a consequence of succinate-induced changes in mean arterial blood pressure (MAP) through RAS activation, rather than a direct effect of succinate through GPR91 in the heart. It was found that MAP was unaffected after two days of succinate treatment, but slightly increased at day 4, and reverted to normal values on the final day of the experiment indicating that the observed hypertrophy induced by succinate was therefore not entirely due to an increase in MAP, but could involve other mechanisms as well [15]. Moreover, echocardiography experiments in rodents, in the presence or absence of losartan, which is an antagonist for the angiotensin II receptor AT-1, demonstrated that losartan abolished succinate-induced increase in blood pressure but did not alter the most relevant echocardiographic parameter for measuring hypertrophy (left ventricular posterior wall thickness - LVPW). These findings were consistent with previous results that showed that succinate could induce RAS activation, but also indicated that succinate-induced hypertrophy was not solely caused by variations in MAP. To connect cardiac hypertrophy to succinate-GPR91 activation, experiments with GPR91-KO mice were performed and showed an increase in left ventricular posterior wall only in wild type mice, but not in GPR91-KO mice. These findings demonstrated that GPR91 is essential for succinate-induced cardiomyocyte hypertrophy. Taken together, these results show that upholding levels of serum succinate can cause cardiac hypertrophy through direct activation of GPR91. However, the fact that losartan reversed some of the other observed hypertrophic effects (besides LVPW) also suggests that succinate-induced remodeling of cardiac muscle involves direct effects of GPR91 in cardiac cells, but also succinate effects in other organs.

**Fig. 5:**
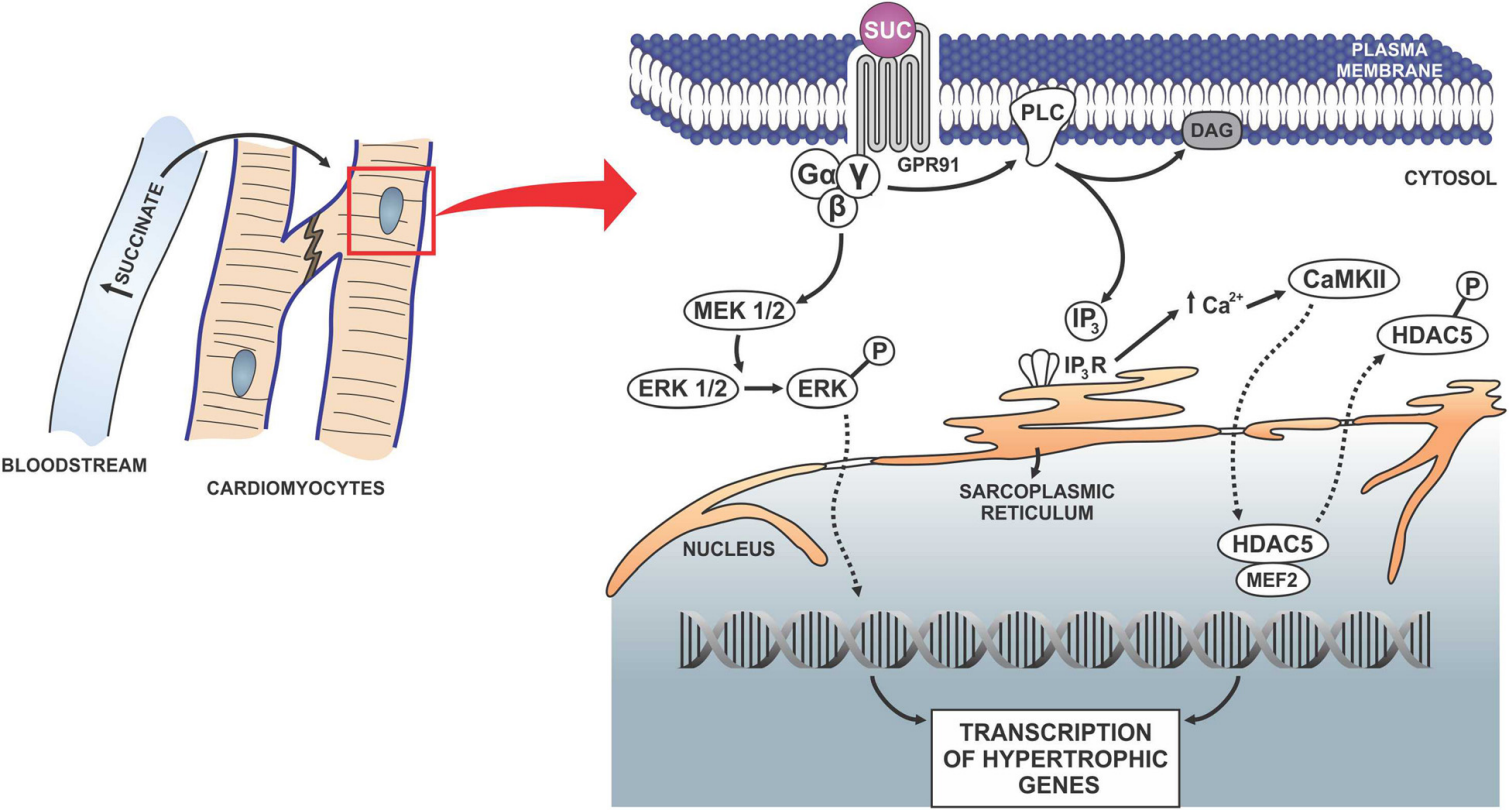
Succinate causes cardiomyocyte hypertrophy via GPR91 activation. Succinate accumulation in the blood stream reaches the cardiac muscle cells to activate GPR91, consequently triggering at least two separate intracellular signaling pathways. In one pathway, GPR91 stimulates MEK1/2 that phosphorylates ERK1/2. Phosphorylated ERK1/2 is translocated to the nucleus, where it activates transcription of hypertrophic genes. In an alternative pathway, GPR91 activates PLC, which produces inositol-3-phosphate and diacylglycerol. IP3 binds to its receptor, releasing Ca^2+^ from the sarcoplasmic reticulum to the cytosol. Ca^2+^ activates CaMKIIδ that moves to the nucleus and phosphorylates HDAC5, which is released from transcription factor MEF2 allowing the transcription of hypertrophic genes

The intracellular signaling events by which GPR91 activation causes cardiac hypertrophy were thus established, using primary culture of neonatal cardiomyocytes [15]. It was shown that upon binding to GPR91, succinate causes CaMKIIδ and ERK1/2 activation, culminating in the transcription of genes related to cardiac hypertrophy [15], (Fig. 5). Succinate/GPR91 activates phospholipase C, which generates inositol 3,4,5-triphosphate (IP_3_) and triggers intracellular Ca^2+^ release from the endoplasmic reticulum (ER). Cytoplasmic Ca^2+^ then activates CaMKIIδ, which phosphorylates histone deacetylase 5 (HDAC5), translocating it out of the nucleus, facilitating the transcription of hypertrophic genes (Fig. 5). Additionally, GPR91 activates MAPK, which phosphorylates ERK1/2. Phosphorylated ERK1/2 (pERK 1/2) translocates to the nucleus, where it also induces cardiac hypertrophy through the aforementioned hypertrophic cellular signaling cascades in cardiomyocytes. Other mechanisms, still not explored, might likewise be involved in succinate/GPR91-induced cardiomyocite hypertrophy.

In addition to these findings in rodent models, it was also demonstrated that humans that underwent a recent ischemic event during organ transplantation, have increased serum succinate levels, and, accordingly, such patients have high levels of blood hypertrophic markers [15]. The clinical applications of these data still need to be explored, but this preliminary finding suggests that succinate could be used as a predictive marker for hypertrophy, and could also be a possible treatment target to eliminate post-ischemic cardiac hypertrophy, often observed after organ transplant.

In this aspect, pioneer systematic structure-activity relationship studies have already identified compounds able to abolish GPR91 function both in human and rats [13]. A compound known as “4c” showed the best antagonic effect *in vitro* (IC_50_ = 7nM), and also regulated in almost 80 % mean arterial blood pressure variation caused by succinate. Different compounds with similar structure, referred to as “5g” and “7e”, remained active after oral intake, a positive result for future pharmacological use. A remarkable fact about theses antagonists is that none of their structures resemble succinate. Given that, the binding mechanism is hitherto not fully understood [12].

## GPR91 signaling in the kidneys and its effects on blood pressure

Besides acting in the heart to contribute to ventricular hypertrophy, succinate can be a potent modulator of renin release. Previous findings by He et al. in 2004, encountered elevated blood pressure in mice treated with succinate. However, the mechanism that led to such increase had not yet been found [11]. Recent work by Vargas et al. in 2009 has finally elucidated the way in which this increase in MAP happens in succinate-treated mice. The experiments showed that accumulation of tubular succinate in the kidneys can activate Macula Densa (MD) cells and cells in the juxtaglomerular apparatus (JGA) to release renin, which is a known pathway for increasing blood pressure via vasoconstriction of peripheral arteries [17]. In this pathway, succinate binds to its receptor GPR91 in the apical region of the MD cells, activates p38 and pERK1/2, which in turn increases COX-2 activity and leads to PGE2 secretion. This release stimulates the cells in the JGA to produce and release renin, acting through the EP2/4 prostaglandin receptors and cAMP, thus activating the known renin-angiotensin system (RAS). This modulation of blood pressure also happens in endothelial cells in the kidney, especially in the afferent arteriole and the glomerular vasculature, where succinate acts through the highly expressed GPR91, increasing Ca^2+^ transient and releasing PGE2, PGI2 (prostacyclin 2) and NO (nitric oxide), which causes renin release in the JGA [19]. A recent microperfusion study has shown that perfusion of the glomeruli with a succinate buffer induces renin release from the JGA and vasodilation of the glomerular afferent arterioles, which abides by the affirmation that succinate has an essential role in glomerular hyperfiltration and RAS activation.

These data suggest that succinate not only has a very important role in blood pressure regulation in ischemic events, but could also be a possible target for novel therapies to control hypertension. For example, post-ischemic transplanted patients could receive GPR91 antagonists to prevent a possible increase in MAP during recovery. That strategy, combined with known treatments for prevention of post operational hypertension, could reduce the cardiovascular risk for other chronical conditions such as arteriosclerosis and aneurisms, both implicated in long-term increases in blood pressure.

## Perpectives in the succinate/GPR91 field

Some evidence has pointed succinate as an ‘alarming’ signal able to trigger GPR91 to sense immunological danger, causing increased allograft rejection during organ transplantation [21]. As described above, blood level of succinate is increased in the serum of patients after liver transplant [15]. Also, a deficiency on mitochondrial succinate cytochrome C reductase, a genetic condition that leads to an increase in succinate levels on serum, is reported as a need for special attention in transplant procedures, since the donor could possibly transfer this inherited metabolic disorder to the recipient [34]. This is a fundamental concern when the donor has parental consanguinity, an increasing factor for autosomal recessive illnesses such as the above-mentioned one. In addition, a multiorgan failure has been reported in a liver-intestine transplant from a pediatric donor with a succinate- cytochrome C-reductase deficiency, a condition that would lead to an increase in blood succinate level. It has also been demonstrated that a patient with succinate dehydrogenase deficiency, another condition that also leads to extracellular accumulation of succinate, exhibited congestive heart failure [35]. Then, GPR91 antagonism in preservation solution for transplantation could represent, for instance, a real benefit to help preventing rejection or other complications.

Thereby, succinate may be a clinical marker for ischemia, and its increase in blood level due to organ transplant should be avoided.

## Conclusions

Since the discovery and characterization of GPR91 - a G-protein couple receptor for succinate - a decade ago, succinate signaling through GPR91 increased our understanding of body functions. It is worth to mention that succinate is now more than sole particpant as a metabolic intermediate. It is known that circulating succinate, through GPR91 activation, is extensively involved in several physio-pathological functions, as shown along this review. Thus, succinate may be considered a signaling molecule with a hormone-like function. Since blood succinate level increase mainly due to ischemia/reperfusion, an emerging application during organ transplantation becomes evident. Therefore, understanding better the participation of Krebs cycle intermediates, as signaling molecules, and their relative contribution to each physiological process in which they might be involved, may lead to a clearer understanding of cell function, and to more accurate clinical interventions when necessary.

## Abbreviations

AT-1: Angiotensin II receptor type 1
CaMKIIδ: Calcium Calmodulin Kinase II δ
cAMP: Cyclic adenosine monophosphate
COX-2: Cyclooxygenase 2
DC: Dendritic cell
EP2/4: Prostaglandin receptors 2/4
ER: Endoplasmic Reticulum
ERK1/2: Extracellular-signal-regulated kinases 1/2
GABA: γ-aminobutyric acid sucnr1
GPR91: G protein-coupled receptor 91
GPR99: G protein-coupled receptor 99
HDAC5: Histone Deacetylase 5
HIF1a: Hypoxia induced factor 1a
HSC: Hepatic stellate cell
IP3: Inositol 3,4,5-triphosphate
JGA: Juxtaglomerular apparatus
JNK: c-Jun N-terminal kinases
LVPW: Left ventricular posterior wall
MAP: Mean arterial blood pressure
MD: Macula densa
PDGF: Platelet-derived growth factor
PERK 1/2: Phosphorylated extracellular signal regulated kinase 1/2
PGE2: Prostaglandin E2
PGI2: Prostacyclin 2
PKA: Protein kinase A
CTAL: Cortical thick ascending limb
RAS: Renin-angiotensin system
RGC: Root ganglion cell
siRNA: Small interference ribonucleic acid
SUCNR: Succinate receptor
TGFβ: Transforming growth factor beta
VEGF: Vascular endothelial growth factor
VO2: Oxygen consumption
WT: Wild-type
